# Efficient yeast ChIP-Seq using multiplex short-read DNA sequencing

**DOI:** 10.1186/1471-2164-10-37

**Published:** 2009-01-21

**Authors:** Philippe Lefrançois, Ghia M Euskirchen, Raymond K Auerbach, Joel Rozowsky, Theodore Gibson, Christopher M Yellman, Mark Gerstein, Michael Snyder

**Affiliations:** 1 Department of Molecular, Cellular and Developmental Biology, Yale University, New Haven, CT 06520, USA; 2 Program in Computational Biology and Bioinformatics, Yale University, New Haven, CT 06520, USA; 3 Department of Molecular Biophysics and Biochemistry, Yale University, New Haven, CT 06520, USA

## Abstract

**Background:**

Short-read high-throughput DNA sequencing technologies provide new tools to answer biological questions. However, high cost and low throughput limit their widespread use, particularly in organisms with smaller genomes such as *S. cerevisiae*. Although ChIP-Seq in mammalian cell lines is replacing array-based ChIP-chip as the standard for transcription factor binding studies, ChIP-Seq in yeast is still underutilized compared to ChIP-chip. We developed a multiplex barcoding system that allows simultaneous sequencing and analysis of multiple samples using Illumina's platform. We applied this method to analyze the chromosomal distributions of three yeast DNA binding proteins (Ste12, Cse4 and RNA PolII) and a reference sample (input DNA) in a single experiment and demonstrate its utility for rapid and accurate results at reduced costs.

**Results:**

We developed a barcoding ChIP-Seq method for the concurrent analysis of transcription factor binding sites in yeast. Our multiplex strategy generated high quality data that was indistinguishable from data obtained with non-barcoded libraries. None of the barcoded adapters induced differences relative to a non-barcoded adapter when applied to the same DNA sample. We used this method to map the binding sites for Cse4, Ste12 and Pol II throughout the yeast genome and we found 148 binding targets for Cse4, 823 targets for Ste12 and 2508 targets for PolII. Cse4 was strongly bound to all yeast centromeres as expected and the remaining non-centromeric targets correspond to highly expressed genes in rich media. The presence of Cse4 non-centromeric binding sites was not reported previously.

**Conclusion:**

We designed a multiplex short-read DNA sequencing method to perform efficient ChIP-Seq in yeast and other small genome model organisms. This method produces accurate results with higher throughput and reduced cost. Given constant improvements in high-throughput sequencing technologies, increasing multiplexing will be possible to further decrease costs per sample and to accelerate the completion of large consortium projects such as modENCODE.

## Background

Novel high-throughput DNA sequencing technologies have allowed the generation of millions of short reads and have empowered a wide variety of studies such as genome-wide analysis of transcriptomes (RNA-Seq) [1-4], transcription factor binding sites (ChIP-Seq) [5,6] and whole-genome sequencing and analysis [7,8]. However, these studies have often been limited by a high cost per sample and low throughput. A typical sequencing run on an Illumina Genome Analyzer II currently costs about $500 in reagents per flowcell lane and requires ~4 days to complete both the sequencing and Illumina analysis pipeline phases. Moreover, the number of mapped reads (up to 10 M per lane) is often significantly higher than required for the experiment, especially for organisms of small genome sizes such as yeasts, worms and flies.

Multiplex DNA sequencing has been pursued since the beginning of Sanger sequencing [9] and has been applied to Roche's 454 platform [10]. Here we describe a multiplexing strategy for Illumina sequencing to process multiple DNA samples simultaneously. The strategy was applied to analyze the targets of three yeast DNA binding proteins (Cse4, Ste12 and RNA polymerase II) using chromatin immunoprecipitation (ChIP) and was shown to yield accurate and high quality results. We also included a reference sample for ChIP-Seq termed input DNA.

ChIP followed by high-throughput sequencing (ChIP-Seq) has been developed to map the protein-DNA interactions at a genome-wide level [5,6]. It allows characterization of transcription factor binding and other DNA-binding proteins during development [5,11], under different environmental conditions [6,12] or in different cell types or tissues. ChIP-Seq has also been used to study the epigenome by mapping the distribution of histone modifications and chromatin-modifying complexes [12-14]. Combination of multiple ChIP-Seq experiments can help to determine transcriptional networks [15].

Cse4 is a centromeric variant of histone H3 [16] and its human homolog is the centromeric protein A (CENP-A) [17]. Yeast centromeres span 126 base pairs and are divided in three centromeric DNA elements (CDEI, CDEII and CDEIII); Cse4 binds CDEII [18]. Cse4 participates in the formation of a specialized hexameric nucleosome with histone H4 and Scm3 that diverges from the standard H2A-H2B-H3-H4 octamer [19-22]. The kinetochore assembles at the centromere and Cse4 is required for normal kinetochore assembly and function [23-26]. Cse4 mutants display strong chromosome missegregation due to incorrect kinetochore structure [25,27,28].

Ste12 is a transcription factor that regulates two sets of genes: those involved in invasive growth (pseudohyphal growth) and those implicated in the mating response (pheromone stimulation) [29-31]. Pseudohyphal growth is a polarized invasion of media by *S. cerevisiae *upon nitrogen starvation. It integrates signals from a MAP kinase cascade and the cAMP-dependant pathway [30,32-35]. During pseudohyphal growth, Ste12 associates as a dimer with Tec1 on transcription factor binding sites (TFBS) upstream of invasive genes [33,36-38]. During the mating response, Tec1 is phosphorylated by Fus3 upon pheromone stimulation, leading to its degradation and the binding of Ste12 on the pheromone response elements [39-41]. Thus, the dual role of Ste12 depends mainly on different phosphorylation events [42-46].

RNA polymerase II (PolII) transcribes most protein-coding genes and is conserved among metazoans. It is recruited to a particular transcription start site depending on the chromatin structure and the presence of preinitiation complexes and transcription factors. In budding yeast, the PolII holoenzyme consists of 12 subunits (Rpb1-12) [47]. Rpb5, Rpb6, Rpb8, Rpb10 and Rpb12 are shared with the two other major RNA polymerases and Rpb5 contacts DNA to promote transcriptional activation [48,49]. PolII directly interacts with the mediator complex, histones, transcription factors, elongation factors and many other proteins [50]. Although yeast RNA polymerase III genome-wide distribution has been studied extensively using microarrays [51-53], only one similar study has been performed to characterize PolII distribution across the yeast genome [54].

Using multiplex ChIP-Seq, we mapped the targets of Cse4, Ste12 and PolII across the yeast genome. We found a large number of binding sites for each factor. Cse4 localized predominantly to centromeres and we also observed other Cse4 binding sites with lower signal intensity elsewhere in the genome. Ste12 and PolII binding sites were found in close proximity to genes.

## Results and discussion

**Figure 1:**
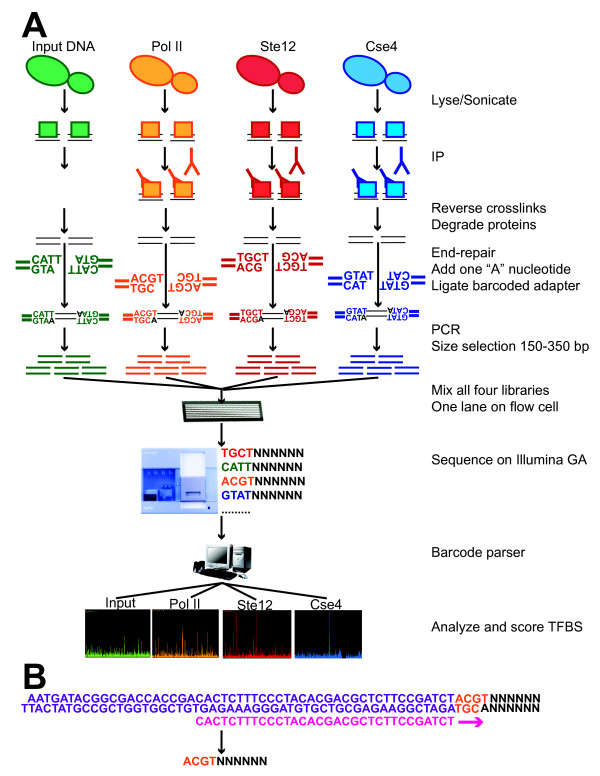
**Scheme for yeast barcoded ChIP-Seq**. (a) Barcoded ChIP-Seq workflow. Ovals depict yeast cells and squares depict proteins. An aliquot of sheared cell lysate is not immunoprecipitated but is otherwise processed normally (green). This DNA, termed input DNA, is a reference sample for ChIP-Seq. Illumina DNA libraries are generated from both ChIP and input DNA samples. In multiplex ChIP-Seq, a barcoded adapter is ligated to an individual DNA sample. The barcode has 3 unique bases followed by a 'T' to anneal with the end-repaired DNA. Four libraries are then pooled together and applied to a single flowcell lane. After sequencing on the Genome Analyzer, reads are separated according to the first four bases and aligned to the yeast genome. Reads are stacked to generate a signal profile and scored against a pool of input DNA to determine significant transcription factor binding sites. (b) The barcode (orange) is located between Illumina adapter sequences (purple) and ChIP or input DNA inserts (black). The sequencing primer (pink) anneals to the adapter sequences and short reads start with the four bases of the barcode (orange) followed by DNA inserts (black). For the sequencing primer and Illumina adapter, oligonucleotide sequences were given by the manufacturer ^© ^2006 Illumina, Inc. All rights reserved.

The barcoded ChIP-Seq workflow is illustrated in Figure 1. We first generated standard Illumina genomic DNA adapter sequences augmented with one of four barcodes (ACGT, CATT, GTAT and TGCT; Table 1). The first three bases uniquely tag or "barcode" a given sample preparation and are separated by a Hamming distance [55] of three which will prevent one barcode being miscalled as another barcode when allowing for one- or two-base sequencing errors. The final 'T' at the fourth base anneals with the 'A' overhang from the end-repaired DNA sample for ligation of ChIP or other DNA fragments. Illumina PCR primers for genomic DNA are used without any changes after adapter ligation to generate the sequencing libraries. Four libraries are pooled together in equimolar ratios and sequenced in a single lane. After sequencing, four distinct sequencing profiles are obtained by parsing the barcodes into distinct bins for subsequent analyses.

**Table 1:** Oligonucleotide sequences for generation of the four barcoded adapters

**Oligo name**	**Barcode**	**Modification**	**Sequence (5'→3')**
MPLEXA1F	GTAT	None	ACACTCTTTCCCTACACGACGCTCTTCCGATCTGTAT
MPLEXA1R	GTAT	5' phosphate	TACAGATCGGAAGAGCTCGTATGCCGTCTTCTGCTTG
MPLEXA6F	CATT	None	ACACTCTTTCCCTACACGACGCTCTTCCGATCTCAT*T*
MPLEXA6R	CATT	5' phosphate	ATGAGATCGGAAGAGCTCGTATGCCGTCTTCTGCTTG
MPLEXA8F	ACGT	None	ACACTCTTTCCCTACACGACGCTCTTCCGATCTACG*T*
MPLEXA8R	ACGT	5' phosphate	CGTAGATCGGAAGAGCTCGTATGCCGTCTTCTGCTTG
MPLEXA9F	TGCT	None	ACACTCTTTCCCTACACGACGCTCTTCCGATCTTGC*T*
MPLEXA9R	TGCT	5' phosphate	GCAAGATCGGAAGAGCTCGTATGCCGTCTTCTGCTTG

### Similar barcode behavior with the same DNA sample

**Figure 2:**
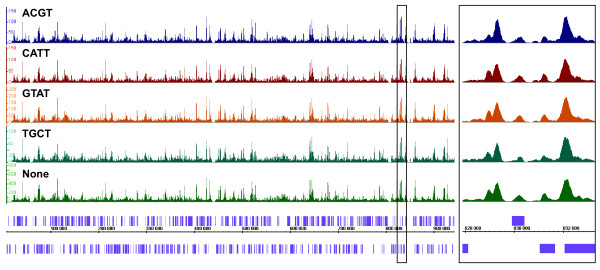
**Comparison of input DNA signal tracks among all four barcoded adapters relative to standard Illumina adapters**. An input sample was split in five aliquots. Four were barcoded differentially (top four lanes) and one had non-barcoded, Illumina adapters (fifth lane, labeled 'None'). Barcoded inputs were scored against non-barcoded input. IGB signal tracks of yeast chromosome 16 are shown for each sample, with ORF locations on the x-axis. ORFs are depicted in purple. On the y-axis, a normalized scale represents the number of read counts at a particular location. Each scale is normalized according to the number of mapped reads (Table 10). A box in the left panel depicts the enlarged section shown in the right panel for positions between 828,000 and 833,000 to demonstrate the overlap among all signal tracks.

To first test whether barcodes generate similar results when added to the same population of DNA fragments, we divided an input DNA sample into five aliquots. The input sample consists of *Saccharomyces cerevisiae *DNA prepared from crosslinked and sonicated chromatin without immunoprecipation and it represents breaks at open chromatin regions (R.K. Auerbach, G.M. Euskirchen, unpublished). Four of the samples were differentially barcoded and the libraries were mixed together in the same flowcell lane. As a control, standard non-barcoded Illumina genomic DNA adapters were added to the fifth aliquot and this library was sequenced in another lane. After sorting reads by barcodes, the barcode sequences were stripped and 26 base pairs were aligned back to the yeast genome. Similar numbers of reads were obtained for each barcoded library. As shown in Figure 2, signal tracks are very similar for the five aliquots. The normalized average tag counts in 500 bp windows are also highly comparable among aliquots (Table 2, R^2 ^= 0.93–0.97). The results for each barcoded library were scored against the control without a barcode to find significant differences; only 36,992 out of 12,156,677 unique bps (0.304%) from the *S. cerevisiae *genome differ significantly for all four barcoded libraries (Figure 2; **See Methods for analysis**). In addition, comparison of peaks among the different libraries revealed high degrees of similarity. These characteristics highly resemble those of a non-barcoded input library analyzed in two different sequencing reactions. Thus, overall biases are not observed in efficiencies of various barcoded libraries and barcoding does not appear to induce differences relative to non-barcoded libraries.

**Table 2:** Comparisons of the average tag count in 500 bp windows across the yeast genome among different samples

Experiment A		Experiment B			
Experiment #	Experiment ID	Experiment #	Experiment ID	R^2^	p-value
10	Input_NB	14	Input_NB	0.9455	< 2.2e^-16^
14	Input_NB	15	Input_ACGT	0.9556	< 2.2e^-16^
14	Input_NB	16	Input_CATT	0.9473	< 2.2e^-16^
14	Input_NB	17	Input_GTAT	0.9685	< 2.2e^-16^
14	Input_NB	18	Input_TGCT	0.9325	< 2.2e^-16^
15	Input_ACGT	16	Input_CATT	0.9422	< 2.2e^-16^
17	Input_GTAT	18	Input_TGCT	0.9304	< 2.2e^-16^

1	PolII_Rep1_ACGT	8	PolII_Rep2_TGCT	0.8904	< 2.2e^-16^
1	PolII_Rep1_ACGT	9	PolII_Rep3_ACGT	0.9104	< 2.2e^-16^
1	PolII_Rep1_ACGT	13	PolII_Rep4_NB	0.9040	< 2.2e^-16^
8	PolII_Rep2_TGCT	9	PolII_Rep3_ACGT	0.9288	< 2.2e^-16^
8	PolII_Rep2_TGCT	13	PolII_Rep4_NB	0.9464	< 2.2e^-16^
9	PolII_Rep3_ACGT	13	PolII_Rep4_NB	0.9302	< 2.2e^-16^

4	Ste12_Rep1_TGCT	7	Ste12_Rep2_GTAT	0.9655	< 2.2e^-16^
4	Ste12_Rep1_TGCT	12	Ste12_Rep3_NB	0.9058	< 2.2e^-16^
7	Ste12_Rep2_GTAT	12	Ste12_Rep3_NB	0.8963	< 2.2e^-16^

Correlation coefficients on the average tag count in 500 bp bins between two experiments among barcoded and non-barcoded input DNA samples from the section "Similar barcode behavior with the same DNA sample" (Comparison 1–7), among barcoded and non-barcoded PolII replicates (Comparison 8–13) and among barcoded and non-barcoded Ste12 replicates (Comparison 14–16). p-values are inferior to 2.2e^-16^, the lowest p-value that R can calculate. See **Methods **for details of the analysis.

### Experimental design for multiplex yeast ChIP-Seq

We next analyzed the distributions of three diverse DNA-binding proteins across the yeast *S. cerevisiae *genome using three to four biological replicates per factor. This study allowed us to 1) further examine the reproducibility of a specified barcode using a different biological replicate 2) determine whether there was crossover signal between barcodes and 3) use the technique to map the binding distributions of three DNA-binding proteins. The proteins selected were the centromeric histone H3 variant Cse4 [16], RNA PolII (each grown in vegetative growth) and the transcription factor Ste12 [31] (incubated in pseudohyphal growth conditions). Cse4 is expected to have a very distinct binding distribution from that of Ste12 which binds near promoters. We also included input DNA as a reference for ChIP-Seq as a fourth sample. Initially we compared three biological replicates for each factor using barcoded sequences for two of the replicates and non-barcoded sequences for the third one. Each biological replicate was made into its own DNA library. The barcoded libraries were pooled and sequenced with one pool per lane (Table 3) whereas the non-barcoded libraries were sequenced in individual lanes. Two lanes were run using the barcoded adapters: Pol II-ACGT, Input-CATT, Cse4-GTAT and Ste12-TGCT (**Pool #1**, Table 3) and Input-ACGT, Cse4-CATT, Ste12-GTAT and PolII-TGCT (**Pool #2**, Table 3). To compare reproducibility of alternative barcodes relative to reproducibility between biological replicates, we sequenced another replicate of Pol II ChIP DNA in a separate pool using the same barcode as one of the other replicates (Pol II-ACGT) (**Pool #3**, Table 3). After sorting reads by barcodes, the barcodes were stripped and 26 base pairs were aligned to the yeast genome. We scored ChIP samples against 8 million input reads derived equally from barcoded and non-barcoded input DNA. Rank-order comparisons between the top targets for two different biological replicates were performed to assess similarity. We obtained 148 targets for Cse4, 823 targets for Ste12 and 2508 targets for PolII (FDR = 0.05; Additional Files 1, 2, 3).

**Table 3:** DNA libraries pooled for multiplex sequencing

**Pool**	**ACGT**	**CATT**	**GTAT**	**TGCT**
**1**	PolII_Rep1	Input_CATT	Cse4_Rep1	Ste12_Rep1
**2**	Input_ACGT	Cse4_Rep2	Ste12_Rep2	PolII_Rep2
**3**	PolII_Rep3	Input_CATT	Cse4_Rep1	Ste12_Rep1

### Comparison of PolII biological replicates

**Figure 3:**
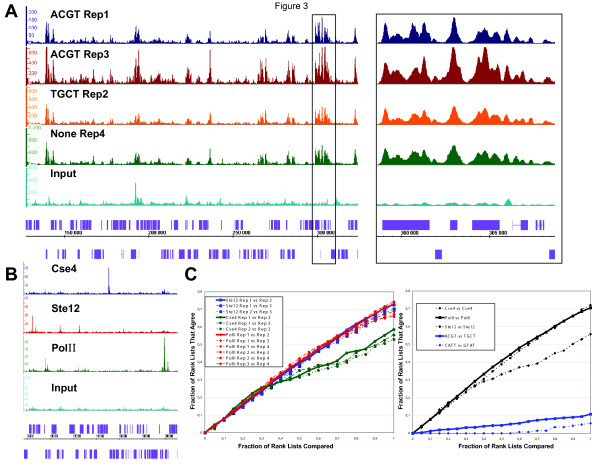
**Barcoded adapters perform similarly to standard Illumina adapters and do not crossover to other samples in the same lane**. (a) RNA PolII binding profiles from different biological replicates with the same barcode (PolII_Rep1, dark blue; PolII_Rep3, red), with different barcodes (PolII_Rep2, orange) or without barcode (PolII_Rep4, green) strongly overlap. See also **Table 3**. Input DNA serves as a reference (light blue). IGB signal tracks of chromosome 5 between 130,000 and 320,000 are shown for each library. A box in the left panel depicts the enlarged section shown in the right panel between positions 298,000 and 309,000 to illustrate the overlap among all PolII signal tracks. (b) Binding profiles from four different libraries pooled and sequenced in the same flowcell lane show very little resemblance. Shown here are the binding profiles for Cse4_Rep2 (dark blue), Ste12_Rep2 (red), PolII_Rep2 (green) and Input_ACGT (light blue). IGB signal tracks of chromosome 12 between 80,000 and 210,000 are shown for each sample. For (a) and (b), axis and scale normalizations are similar to **Figure 2**. (c) Left: Rank-rank comparison of target lists between all pairwise barcoded replicates for Cse4, PolII and Ste12. The horizontal axis shows the fraction of the two lists being compared and the vertical axis shows the fraction of those targets that agree between a given pair of target lists. All comparisons show strong agreement, although the rank lists for Cse4 differ more than PolII or Ste12 for the second half of their length. Right: Rank-rank comparison between barcoded replicates from the same factors (averaged over all pairwise comparisons) compared to rank-rank comparisons for barcoded replicates between different factors: PolII_Rep1 (ACGT) vs. Ste12_Rep1 (TGCT) and Cse4_Rep2 (CATT) vs. Ste12_Rep2 (GTAT).

By comparing two biological replicates of PolII ChIP DNA with the same barcode (ACGT) we found a close correlation between the target lists (Figure 3a and 3c). A similar close correlation was observed by comparing the targets of the individual biological replicates using different barcodes among each of the different factors (Figure 3c). Furthermore, comparison of normalized average tag counts in 500 bp windows between biological replicates for PolII and Ste12 revealed strong correlations (Table 2, R^2 ^= 0.89–0.97). These values are similar to the correlation coefficients between biological replicates found in a recent yeast RNA-Seq study (R^2 ^= 0.87–0.90) [1]. Thus, consistent with barcoded input DNA, the different barcodes give similar results when analyzing similar libraries. Our results strongly suggest that the barcode has negligible impact on binding site detection.

### Comparison of ChIP-Seq data to previously published ChIP-chip data

We also compared the binding site distributions for Ste12, Cse4 and PolII to previously published data [16,36,37,54,56]. Our Ste12 ChIP-Seq targets overlap with 67% (232/346) of the targets found in **Borneman et al. **[37] while our PolII ChIP-Seq targets overlap with 72% (667/929) of the PolII ChIP-chip targets from the **Steinmetz et al. **data [54]. These overlaps between ChIP-Seq and ChIP-chip do not differ significantly from those determined in other studies [6], typically in the 64–71% range. However, we observe 2- to 4-fold more targets using ChIP-Seq as compared to ChIP-chip, consistent with the observation that ChIP-Seq is more sensitive than ChIP-chip [6].

### Non-overlapping binding profiles among samples sequenced simultaneously

By comparing binding profiles obtained from a single lane containing four different barcoded libraries, we observed that many targets belong uniquely to a single signal track and their distributions are consistent with the biological roles of the factors studied (Figure 3b and 3c). This is particularly the case for Ste12 and Cse4 which have highly dissimilar binding patterns and almost no target overlap (Figure 3c). Note that some peaks are present in both ChIP and input samples and are removed after scoring. Thus, analysis of different ChIP samples with distinct barcodes indicates that the signal from one barcoded library does not crossover to those of other barcoded libraries (Figure 3b). We define signal crossover as the erroneous assignment of a barcoded read from a particular ChIP sample to the wrong ChIP sample during multiplex ChIP-Seq, generating a signal profile for one barcoded sample with reads from other ChIPs sequenced simultaneously.

### Ste12, PolII and Cse4 binding distributions

**Figure 4:**
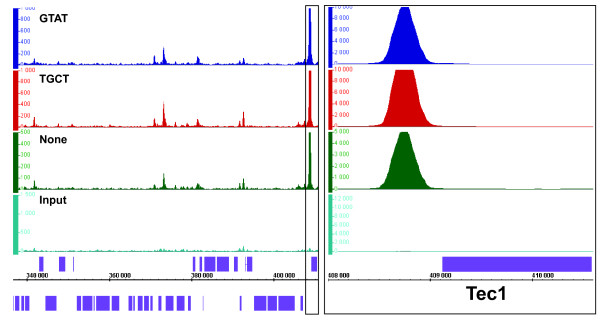
**Ste12 distribution during pseudohyphal growth is similar across three different biological replicates**. Two barcoded replicates (Ste12_Rep2, dark blue; Ste12_Rep1, red) and a non-barcoded replicate (Ste12_Rep3, green) were compared to input DNA (light blue). Ste12 ChIP samples were scored against a pool of input DNA. IGB signal tracks of chromosome 2 between 340,000 and 410,000 are shown for each sample. Axis and scale normalizations are similar to **Figure 2**. A box in the left panel containing the *TEC1 *gene and its surrounding intergenic region was enlarged in panel B and rescaled to emphasize the strong signal at the *TEC1 *promoter. The same normalization as in **Figure 2 **was applied. Ste12p and Tec1p act as a dimer during pseudohyphal growth [31].

**Figure 5:**
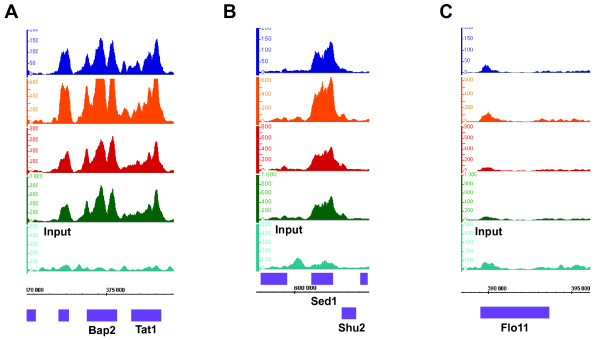
**PolII signal profiles recapitulate findings from Steinmetz et al**. PolII ChIP-Seq signal profiles resemble very closely to those published in Figure 3 of Steinmetz et al [54]. We obtained consistent binding at the Bap2-Tat1 loci (a) and at the Sed1-Shu2 loci (b). As expected, we did not observe binding at the Flo11 locus (c). For PolII ChIP-Seq experiments, two biological replicates were barcoded with ACGT (PolII_Rep1, dark blue; PolII_Rep2, orange), one was barcoded with TGCT (PolII_Rep3, red) and a fourth replicate had non-barcoded adapters (PolII_Rep4, green). Input DNA serves as a reference (light blue). Axis and scale normalizations are similar to **Figure 2**. ORFs above the coordinates axis are on the Watson strand while ORFs below this axis are on the Crick strand.

**Figure 6:**
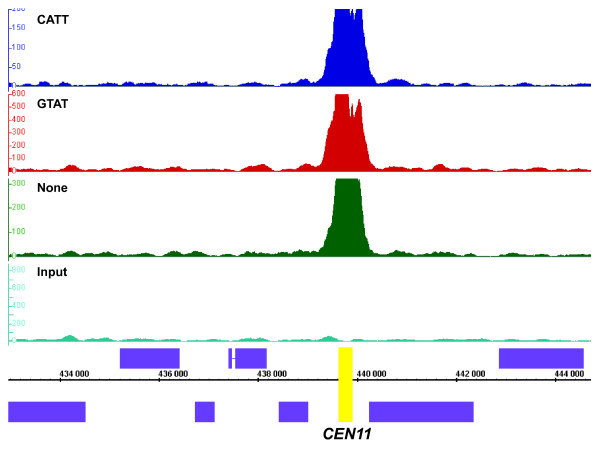
**Cse4p is found robustly at centromeres**. All biological replicates were strongly and tightly bound to centromeres, as it is depicted here in the case of *CEN11*. Two barcoded replicates (Cse4_Rep2, dark blue; Cse4_Rep1, red) and a non-barcoded replicate (Cse4_Rep3, green) were compared to input DNA (light blue). Cse4 ChIP samples were scored against a pool of input DNA. IGB signal tracks of the *CEN11 *on chromosome 11 are shown for each sample. *CEN11 *is highlighted in a yellow box. Axis and scale normalizations are similar to **Figure 2**.

Analysis of the binding targets of Ste12, Pol II and Cse2 reveals expected and novel results. Ste12p binds upstream of genes implicated in pseudohyphal growth such as Tec1, Mga1, Phd1 and Flo8 (Figure 4 and [31,36]). As expected, Ste12 targets are enriched for GO categories including pseudohyphal growth, cell wall organization, and stress response (Table 4 and Additional File 5), which is consistent with the role of Ste12p in regulating the pseudohyphal pathway. The PolII distribution in mid-log phase intersects ~2500 genes, which is fewer than that reported by recent RNA-Seq studies [1]. This may be due to differences in the analysis of ChIP-Seq and RNA-Seq data. Enriched regions in ChIP-Seq data are determined using a threshold for a minimal read count difference at a particular region between the ChIP sample and input DNA while RNA-Seq data is analyzed using a global threshold for a minimal number of mapped reads. Overall, we recapitulated all the binding profiles displayed in the genome-wide PolII ChIP-chip study by **Steinmetz et al. **(Figure 5) [54]. As expected, PolII targets are overrepresented in GO categories such as translation, transcription, RNA processing and primary metabolism, which are crucial for exponential growth in rich media (Table 4 and Additional File 5). Cse4p binds strongly to all yeast 16 centromeres as shown previously (Figure 6 and[16]). Our data indicate that Cse4 tightly occupies a narrow region around the centromere. In addition, we also detected significant binding relative to background at 132 novel non-centromeric sites; Cse4p is less abundant at these sites relative to centromeric regions (Additional File 1). These non-centromeric sites are enriched for metabolic genes, in particular those involved in glucose metabolism (e.g. glycolysis, hexose and glucose catabolism), which are highly expressed under our conditions (Table 4 and Additional File 5). In addition, nearly all non-centromeric Cse4 target genes are present among the 100 highest ranked PolII targets. To explain this novel finding, we hypothesize that Cse4 transiently integrates at regions of high histone turnover which could now be detected using ChIP-Seq. Cse4p might temporarily integrate at these non-centromeric sites before being outcompeted by histone H3 since it has been demonstrated by ChIP-qPCR that a Cse4 mutant protein can localize to non-centromeric locations when the stoichiometry between histone H3 and Cse4p is perturbed [27].

**Table 4:** GO process analysis of gene targets for Cse4, Ste12 and PolII. For a complete GO analysis, please refer to Additional File 5.

**GO term**	**Cluster frequency**	**Background frequency**	**p-value**
GO process categories for Cse4			

glycolysis	8 out of 132 genes, 6.1%	22 out of 7158 background genes, 0.3%	8.32e-07
glucose catabolic process	8 out of 132 genes, 6.1%	32 out of 7158 background genes, 0.4%	2.34e-05
alcohol metabolic process	16 out of 132 genes, 12.1%	179 out of 7158 background genes, 2.5%	4.49e-05
hexose catabolic process	8 out of 132 genes, 6.1%	37 out of 7158 background genes, 0.5%	7.96e-05
cellular biosynthetic process	57 out of 132 genes, 43.2%	1689 out of 7158 background genes, 23.6%	0.00013
monosaccharide catabolic process	8 out of 132 genes, 6.1%	42 out of 7158 background genes, 0.6%	0.00023
alcohol catabolic process	8 out of 132 genes, 6.1%	45 out of 7158 background genes, 0.6%	0.00039
pyruvate metabolic process	7 out of 132 genes, 5.3%	39 out of 7158 background genes, 0.5%	0.00174
hexose metabolic process	9 out of 132 genes, 6.8%	78 out of 7158 background genes, 1.1%	0.00349
biosynthetic process	58 out of 132 genes, 43.9%	1929 out of 7158 background genes, 26.9%	0.00496
hexose biosynthetic process	6 out of 132 genes, 4.5%	31 out of 7158 background genes, 0.4%	0.00529
glucose metabolic process	8 out of 132 genes, 6.1%	64 out of 7158 background genes, 0.9%	0.00601

GO process categories for Ste12			

biological regulation	193 out of 823 genes, 23.5%	1132 out of 7158 background genes, 15.8%	7.46e-07
regulation of cellular process	158 out of 823 genes, 19.2%	889 out of 7158 background genes, 12.4%	2.02e-06
regulation of biological process	160 out of 823 genes, 19.4%	904 out of 7158 background genes, 12.6%	2.13e-06
monosaccharide transport	15 out of 823 genes, 1.8%	24 out of 7158 background genes, 0.3%	3.48e-06
hexose transport	15 out of 823 genes, 1.8%	24 out of 7158 background genes, 0.3%	3.48e-06
multi-organism process	40 out of 823 genes, 4.9%	136 out of 7158 background genes, 1.9%	9.27e-06
response to pheromone	32 out of 823 genes, 3.9%	96 out of 7158 background genes, 1.3%	1.03e-05
response to chemical stimulus	86 out of 823 genes, 10.4%	412 out of 7158 background genes, 5.8%	1.18e-05
response to stimulus	144 out of 823 genes, 17.5%	818 out of 7158 background genes, 11.4%	2.41e-05
cellular carbohydrate metabolic process	47 out of 823 genes, 5.7%	187 out of 7158 background genes, 2.6%	0.00011
growth	40 out of 823 genes, 4.9%	148 out of 7158 background genes, 2.1%	0.00012
regulation of transcription from RNA Pol II	56 out of 823 genes, 6.8%	243 out of 7158 background genes, 3.4%	0.00017
regulation of cell size	34 out of 823 genes, 4.1%	117 out of 7158 background genes, 1.6%	0.00017
sexual reproduction	34 out of 823 genes, 4.1%	120 out of 7158 background genes, 1.7%	0.00033
conjugation	34 out of 823 genes, 4.1%	120 out of 7158 background genes, 1.7%	0.00033
conjugation with cellular fusion	34 out of 823 genes, 4.1%	120 out of 7158 background genes, 1.7%	0.00033
response to abiotic stimulus	32 out of 823 genes, 3.9%	111 out of 7158 background genes, 1.6%	0.00047
cellular structure morphogenesis	40 out of 823 genes, 4.9%	156 out of 7158 background genes, 2.2%	0.00057
anatomical structure development	40 out of 823 genes, 4.9%	156 out of 7158 background genes, 2.2%	0.00057
cell morphogenesis	40 out of 823 genes, 4.9%	156 out of 7158 background genes, 2.2%	0.00057
anatomical structure morphogenesis	40 out of 823 genes, 4.9%	156 out of 7158 background genes, 2.2%	0.00057
signal transduction	51 out of 823 genes, 6.2%	222 out of 7158 background genes, 3.1%	0.00067
carbohydrate metabolic process	49 out of 823 genes, 6.0%	210 out of 7158 background genes, 2.9%	0.00068
external encapsulating structure organization	48 out of 823 genes, 5.8%	206 out of 7158 background genes, 2.9%	0.00093
cell wall organization and biogenesis	48 out of 823 genes, 5.8%	206 out of 7158 background genes, 2.9%	0.00093
carboxylic acid metabolic process	67 out of 823 genes, 8.1%	332 out of 7158 background genes, 4.6%	0.00195
organic acid metabolic process	67 out of 823 genes, 8.1%	332 out of 7158 background genes, 4.6%	0.00195
carbohydrate transport	15 out of 823 genes, 1.8%	35 out of 7158 background genes, 0.5%	0.00254
cell growth	26 out of 823 genes, 3.2%	87 out of 7158 background genes, 1.2%	0.00268
reproduction	65 out of 823 genes, 7.9%	323 out of 7158 background genes, 4.5%	0.00309
hexose metabolic process	24 out of 823 genes, 2.9%	78 out of 7158 background genes, 1.1%	0.00367
glucose metabolic process	21 out of 823 genes, 2.6%	64 out of 7158 background genes, 0.9%	0.00478
pseudohyphal growth	21 out of 823 genes, 2.6%	65 out of 7158 background genes, 0.9%	0.00631
cell communication	56 out of 823 genes, 6.8%	271 out of 7158 background genes, 3.8%	0.00665
nitrogen compound metabolic process	51 out of 823 genes, 6.2%	241 out of 7158 background genes, 3.4%	0.00860
establishment and maintenance of cell polarity	32 out of 823 genes, 3.9%	126 out of 7158 background genes, 1.8%	0.00953

Top 40 GO process categories for PolII			

cellular process	1864 out of 2508 genes, 74.3%	4710 out of 7158 background genes, 65.8%	8.82e-27
ribonucleoprotein complex biogenesis	289 out of 2508 genes, 11.5%	494 out of 7158 background genes, 6.9%	3.36e-25
ribosome biogenesis	246 out of 2508 genes, 9.8%	407 out of 7158 background genes, 5.7%	7.01e-24
cellular component organization and biogenesis	785 out of 2508 genes, 31.3%	1725 out of 7158 background genes, 24.1%	4.02e-22
organelle organization and biogenesis	621 out of 2508 genes, 24.8%	1331 out of 7158 background genes, 18.6%	2.59e-19
primary metabolic process	1276 out of 2508 genes, 50.9%	3187 out of 7158 background genes, 44.5%	1.59e-12
maturation of SSU-rRNA from tricistronic rRNA	49 out of 2508 genes, 2.0%	60 out of 7158 background genes, 0.8%	1.86e-10
RNA metabolic process	486 out of 2508 genes, 19.4%	1082 out of 7158 background genes, 15.1%	2.71e-10
nucleotide and nucleic acid metabolic process	649 out of 2508 genes, 25.9%	1512 out of 7158 background genes, 21.1%	6.60e-10
maturation of SSU-rRNA	49 out of 2508 genes, 2.0%	62 out of 7158 background genes, 0.9%	1.97e-09
rRNA metabolic process	143 out of 2508 genes, 5.7%	257 out of 7158 background genes, 3.6%	7.39e-09
ribosomal large subunit biogenesis	50 out of 2508 genes, 2.0%	65 out of 7158 background genes, 0.9%	7.46e-09
ribonucleoprotein complex assembly	96 out of 2508 genes, 3.8%	156 out of 7158 background genes, 2.2%	1.23e-08
macromolecule metabolic process	1096 out of 2508 genes, 43.7%	2752 out of 7158 background genes, 38.4%	1.71e-08
biopolymer metabolic process	1033 out of 2508 genes, 41.2%	2580 out of 7158 background genes, 36.0%	2.55e-08
ribosomal subunit assembly	44 out of 2508 genes, 1.8%	56 out of 7158 background genes, 0.8%	4.08e-08
cellular localization	303 out of 2508 genes, 12.1%	644 out of 7158 background genes, 9.0%	4.38e-08
ribosome assembly	49 out of 2508 genes, 2.0%	65 out of 7158 background genes, 0.9%	4.46e-08
cellular metabolic process	1340 out of 2508 genes, 53.4%	3448 out of 7158 background genes, 48.2%	4.55e-08
metabolic process	1356 out of 2508 genes, 54.1%	3496 out of 7158 background genes, 48.8%	6.00e-08
rRNA processing	137 out of 2508 genes, 5.5%	250 out of 7158 background genes, 3.5%	9.13e-08
ncRNA processing	172 out of 2508 genes, 6.9%	335 out of 7158 background genes, 4.7%	3.92e-07
cellular macromolecular complex organization	184 out of 2508 genes, 7.3%	364 out of 7158 background genes, 5.1%	4.79e-07
establishment of localization in cell	278 out of 2508 genes, 11.1%	594 out of 7158 background genes, 8.3%	6.51e-07
RNA processing	234 out of 2508 genes, 9.3%	487 out of 7158 background genes, 6.8%	9.22e-07
gene expression	704 out of 2508 genes, 28.1%	1708 out of 7158 background genes, 23.9%	9.27e-07
intracellular transport	260 out of 2508 genes, 10.4%	551 out of 7158 background genes, 7.7%	9.36e-07
regulation of translation	38 out of 2508 genes, 1.5%	49 out of 7158 background genes, 0.7%	1.88e-06
maturation of 5.8S rRNA	37 out of 2508 genes, 1.5%	48 out of 7158 background genes, 0.7%	4.22e-06
maturation of 5.8S rRNA from tricistronic rRNA	37 out of 2508 genes, 1.5%	48 out of 7158 background genes, 0.7%	4.22e-06
posttranscriptional regulation of gene expression	44 out of 2508 genes, 1.8%	61 out of 7158 background genes, 0.9%	4.99e-06
macromolecular complex subunit organization	197 out of 2508 genes, 7.9%	404 out of 7158 background genes, 5.6%	5.30e-06
nuclear export	64 out of 2508 genes, 2.6%	102 out of 7158 background genes, 1.4%	1.28e-05
localization	448 out of 2508 genes, 17.9%	1046 out of 7158 background genes, 14.6%	1.35e-05
nuclear transport	76 out of 2508 genes, 3.0%	128 out of 7158 background genes, 1.8%	1.86e-05
nucleocytoplasmic transport	76 out of 2508 genes, 3.0%	128 out of 7158 background genes, 1.8%	1.86e-05
cleavages during rRNA processing	29 out of 2508 genes, 1.2%	36 out of 7158 background genes, 0.5%	3.89e-05
RNA 5'-end processing	26 out of 2508 genes, 1.0%	31 out of 7158 background genes, 0.4%	4.30e-05
transcription from RNA polymerase I promoter	30 out of 2508 genes, 1.2%	38 out of 7158 background genes, 0.5%	5.25e-05
regulation of cellular biosynthetic process	46 out of 2508 genes, 1.8%	68 out of 7158 background genes, 0.9%	5.66e-05

GO process analysis (p < 0.01) for Cse4 combined targets, for Ste12 combined targets and for PolII combined targets.

For a complete GO analysis, please refer to Additional File 5.

### Artifacts absent in barcoded libraries but present in non-barcoded libraries

Finally, we did observe one set of differences between barcoded and non-barcoded libraries. A curious rare artifact was found in non-barcoded libraries and excluded in barcoded libraries. ~31 abnormally-shaped peaks were present in non-barcoded ChIP libraries in which the same sequences was obtained many times resulting in block-shaped peaks (Additional File 4). This artifact was not observed in barcoded libraries.

Although the source of this artifact is not clear, it might be due to specific priming from the non-barcoded adaptor on yeast DNA. Regardless, these artifacts are readily eliminated by removing non-unique reads. We did not find additional areas of dissimilarity between barcoded and non-barcoded libraries.

## Conclusion

Our multiplex strategy for ChIP-Seq allows reliable and accurate analysis of multiple samples simultaneously. We processed four times as many samples in the same amount of time and reduced our cost per sample by 65% (Table 5). Barcoded ChIP-Seq will greatly facilitate large-scale studies in organisms with large genomes for which smaller numbers of reads are required and will be particularly valuable for organisms with small genomes such as bacteria, viruses, other yeasts, algae such as *Chlamydomonas reinhardtii *and plants such as *A. thaliana*. The relatively small genomes of two model organisms, the worm *C. elegans *and the fruit fly *D. melanogaster*, are under intensive characterization by the modENCODE consortium [57]. modENCODE (Model organism ENCyclopedia Of DNA Elements) aims to identify all functional elements, including transcription factor binding sites and transcribed regions, in the genomes of *C. elegans *and *D. melanogaster*. Thus the application of barcoded ChIP-Seq to globally map transcription factor binding sites in *C. elegans *and *D. melanogaster *will be valuable for cost-effective completion of this project. We estimate for yeast that we would need 260,000, 90,000 and 18,000 mapped reads to saturate transcription factor binding sites, given fold-enrichments of 5×, 10× and 50× respectively (Table 6). With an enrichment of 10-fold, 800,000 mapped reads would be needed for *C. elegans*, 1,000,000 reads for *D. melanogaster *and 1,500,000 reads for *A. thaliana *(Table 7). These numbers can be easily achieved with barcoded ChIP-Seq. Moreover, considering the high degree of ChIP-Seq target overlap between different biological replicates (Figure 3c), we estimate that two biological replicates are sufficient to achieve statistical significance for a particular ChIP-Seq experiment if the biological replicates are very similar. Otherwise, three biological replicates should be used if there is a higher level of divergence among different biological replicates.

**Table 5:** Cost per sample and number of mapped reads with increasing multiplexing

**Number of barcoded adapters**	Fraction of non-barcoded cost per sample*	Number of mapped reads**
1	100%	10.00 M
4	34.61%	2.50 M
8	23.71%	1.25 M
12	20.08%	0.83 M
16	18.26%	0.63 M
40	15.00%	0.25 M

*Includes Illumina DNA library preparation (fixed cost), sequencing run

*Excludes ChIP reagents, Illumina GAII sequencer, HR and data storage

*For a non-barcoded sample, the cost per sample is $498.23 USD as of October 2008.

**Assumes optimal mapping values for a lane run on a GA II (10 million mapped reads)

**Table 6:** ChIP-Seq simulations of depth of sequencing for *S. cerevisiae*

Fold Enrichment	2×	5×	10×	50×
Number of Reads	2,500,000	260,000	90,000	18,000

Number of reads required in order to identify greater than 95% of 600 simulated binding sites distributed evenly over the 12 Mb *S. cerevisiae *genome with average fold enrichments of 2×, 5×, 10× and 50×. See **Methods **for details of the simulation.

**Table 7:** ChIP-Seq simulations of depth of sequencing for three model organisms

		Fold Enrichment of Simulated Sites	
	Genome Size (Mb)	5×	10×
*C. elegans*	100	2,400,000	800,000
*D. melanogaster*	123	3,500,000	1,000,000
*A. thaliana*	157	6,800,000	1,500,000

Number of reads required in order to identify greater than 95% of 1,000 simulated binding sites distributed over the genomes of *C. elegans*, *D. melanogaster *and *A. thaliana *for average fold enrichments of 5× and 10×. See **Methods **for details of the simulation.

Our general multiplexing approach is expected to be useful for a variety of other applications including bacterial genome re-sequencing and RNA-Seq; it will also be valuable for other short read DNA sequencing platforms. Using the current technology, genome re-sequencing in yeasts, with a 25× coverage for SNP detection, is not amenable to multiplexing as it would require about 9.50 M mapped reads of length 32 (36 bp minus 4-bp barcode). This is equivalent to one or two Illumina flowcell lanes. RNA-Seq experiments could also be multiplexed in the future although considerable sequencing depth is required to detect at a significant level low-abundance transcripts or rare splice variants. In general, to detect binding sites or transcripts that are present in low abundance by barcoded ChIP-Seq or RNA-Seq, it is important to determine by calculations or computer simulations the minimal number of mapped reads per sample to establish the appropriate level of multiplexing. With the new Illumina Genome Analyzer II, there is an increase in the number of reads that can be obtained (currently ~15 million per lane) and it will be possible to further multiplex ChIP-Seq by creating many other different barcoded adapters, ligating them to individual samples and pooling all of these barcoded libraries in the same flow cell lane. Sequencing reagent costs per sample would further decrease from 65% (4 barcodes) to 76% (8 barcodes), 80% (12 barcodes), 82% (16 barcodes) and 85% (40 barcodes), in comparison to non-barcoded samples (Table 5).

To maintain base balance at any given sequencing cycle, indexing adapters should be designed in increments of four. We prefer to keep the barcodes distinct from one another to avoid crossover due to sequencing errors; for a 3-base barcode this leads to 16 different combinations. To increase multiplexing beyond this figure one can either use barcodes with one base difference and correct for sequencing errors or increase the size of the barcode. Massively barcoded ChIP-Seq, bearing constant improvements in ultra-throughput sequencing technologies, will become widely accessible and is expected to supplant array-based technologies for gene regulation and expression studies in organisms with both small and larger genomes.

## Methods

### Data accession and supplementary methods

The data discussed in this publication have been deposited in NCBI's Gene Expression Omnibus [58] and are accessible through GEO Series accession number GSE13322 [59]. Sgr files and other data can be accessed at this URL address: 

### Cell growth

Yeast strains are described in Table 8. Yeast strains CMY288-1B, CMY8058-3-4 and CMY8082-20-3 were grown in YPAD rich media to exponential mid-log phase (OD_600 _= 1.0, 500 mL culture). For yeast strains YJM339 and CMY291, cells were grown overnight to mid-log phase to OD_600 _= 0.6 and then pseudohyphal growth was conducted in nitrogen-depleted SLAD for 4 h as described [36].

**Table 8:** Yeast strains used in this study

**Strain ID**	**Genotype**	**Parent strain**	**Source**
CMY 288-1B	MATα his3Δ1 leu2Δ0 lys2Δ0 ura3Δ0	BY 4741	Christopher Yellman, unpublished
YJM 339	MATa/α HO/HO	Clinical isolate	McCusker et al., 1994
CMY 291	MATa/α HO/HO STE12-13MYC-kanMX6/STE12-13MYC-kanMX6	YJM 339	Christopher Yellman, unpublished
CMY 8058-3-4	MATa ade2-1 bar1::loxP can1H cyh2H gal1H his3Δ1 leu2-3,112 trp1-289 ura3-52	A364a	Christopher Yellman, unpublished
CMY 8082-20-3	MATa ade2-1 bar1::loxP can1H cyh2H gal1H his3Δ1 leu2-3,112 trp1-289 ura3-52 CSE4-3HA-URA3	A364a	Christopher Yellman, unpublished; Meluh et al., 1998

### Chromatin immunoprecipation (ChIP)

Chromatin immunoprecipitations were performed as described [60]. All ChIP experiments were completed as biological triplicates for Cse4 and Ste12 and as biological quadruplicates for PolII. For the immunoprecipitations of Ste12 and Cse4, Ste12-13X Myc (160 uL of a 50% anti-Myc EZiew affinity gel; Sigma) and Cse4-3HA (160 uL of a 50% anti-HA EZiew affinity gel; Sigma) were pre-washed three times in lysis/IP buffer containing protease inhibitors and PMSF and added to the lysates from their respective epitope-tagged strains and untagged controls. For the immunoprecipitation of native RNA polymerase II from strain CMY288-1B, 20 μL of mouse ascites containing RNA polymerase II 8WG16 mouse monoclonal antibody (Covance, Cat. #MMS-126R) was added to one set of triplicates. The other set was left without antibody as a control.

Immunoprecipitations were carried out with inverting at 4°C for 14–16 h. For the PolII samples, 250 uL of a 50% Protein G agarose slurry (washed twice in lysis/IP buffer) was added to each of the PolII samples as well as to the no antibody controls, and incubated for 1 h at 4°C with gentle inverting.

### Input DNA preparation

To isolate input DNA, 250 uL of clarified CMY288-1B lysate was reserved before immunoprecipitation and combined with 250 μL of 1× TE [pH 8.0]/1% SDS. Crosslinks were reversed by an overnight incubation at 65°C and Proteinase K treatment was carried out as described above. Instead of direct DNA precipitation as for ChIP samples, input DNA was extracted three times using phenol:chloroform:isoamyl alcohol (25:24:1) (Fluka) and once with chloroform alone. To precipitate DNA, 50 μL of 5 M LiCl and 1 mL of 100% ethanol were mixed with the upper phase isolated from the chloroform extraction and precipitation occurred for 1 h at -20°C. Samples were centrifuged for 20 min at 14,000 RPM at 4 °C and were air-dried for 10 min. DNA was resuspended in 30 μL 1× TE [pH 8.0] and input DNA was incubated at 37°C for 30 min with 2 μL of DNase-free RNase A. Finally, input DNA was further purified using a Qiagen MinElute column.

### Real-time quantitative polymerase chain reaction

**Figure 7:**
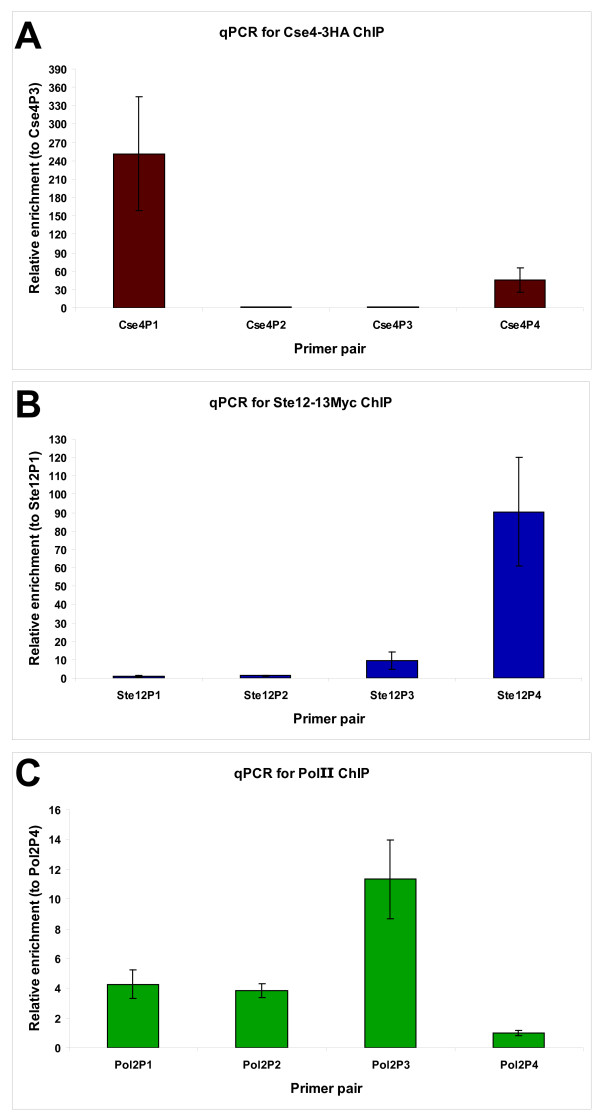
**qPCR analysis for ChIP samples Cse4-3HA (a), Ste12-13Myc (b) and RNA polymerase PolII (c)**. For all qPCR analysis, normalization using the 2^-ΔΔCp ^method was used to compare results from a given primer pair to a negative primer pair (respectively Cse4P2, Ste12P1 and Pol2P4) (**Table 9**). Error bars represent standard deviation across three biological replicates for relative enrichment to the negative primer pair. (a) Cse4p is enriched preferentially at the centromeres (Cse4P1 for *CEN3 *and Cse4P4 for *CEN6*) but not at two random non-centromeric locations (Cse4P2 and Cse4P3). (b) Ste12p binds known target sites in pseudohyphal growth. Ste12P1, Ste12P2, Ste12P3 and Ste12P4 represent respectively sites with no enrichment, low enrichment, medium enrichment and high enrichment as determined by ChIP-chip studies (Christopher M. Yellman, unpublished data). While the low enrichment site was not found to be significantly enriched for this ChIP sample, the medium and high enrichment sites were strongly present in our samples used for qPCR. (c) PolII primer pairs were selected using Steinmetz microarray data [54,64] to have three positive pairs (Pol2P1, Pol2P2 and Pol2P3) and one negative pair (Pol2P4). Positive targets were all significantly enriched over the negative control.

Real time quantitative PCR (qPCR) with SYBR green dye was performed using a Roche LightCycler 480 qPCR machine to confirm enrichment of control regions. A two-fold enrichment between experimental samples (epitope-tagged strains for Ste12 and Cse4, primary antibody for PolII) and reference samples (untagged strains for Ste12 and Cse4, protein G beads only for PolII) for known binding sites was set as the threshold for samples to continue forward towards Illumina library preparation. For each DNA-binding protein studied, four primer pairs were designed and these included at least two primer pairs amplifying known binding sites and at least one primer pair amplifying a random region as a negative control. Primers were generated using Primer3  with the following criteria: fragment size between 200 and 250 bp, primer length 20 and melting temperature between 59°C and 61°C. Other settings were kept as default. Primer sequences are given in Table 9. A standard curve with a dilution series of genomic DNA was generated for each primer pair to determinate primer pair efficiency [61]. Each 10 μL reaction contained 2 μL of nuclease-free water (Gibco), 5 μL of LightCycler 480 SYBR Green I Master mix (Roche, Cat. #04 707 516 001), 0.5 μL of each primer (10 μM stocks) and 2 μL of either water (negative control), diluted genomic DNA (standard curve) or ChIP DNA (1/21 dilution). For ChIP DNA, reactions were done in duplicates on the 384-well plate. Phenol-chloroform extracted genomic DNA was diluted to 8^-1^, 8^-2^, 8^-3^, 8^-4^, 8^-5^, 8^-6 ^and 8^-7 ^to generate a dilution series for determination of the standard curve. Each qPCR reaction was run using the same program: pre-incubation (95°C for 5 min), 45 cycles of amplification (95°C for 20 s, 54°C for 30 s, 72°C for s), melting curves using a heat ramp and cool down. Crossing point values (Cp values) were obtained using the second derivative maximal analysis tool included in the Roche LightCycler480 software. To determine enrichment, the 2^-ΔΔCp ^method was used by comparing enrichment values for positive primer pairs to a negative primer pair between experimental and reference ChIP experiments. The negative primer pair should not give differences between experimental samples and control samples. Average enrichment for all biological replicates was determined for each primer pair and standard deviation to the mean was indicated as error bars. Mean enrichments over negative primer pair (+/- SD) were plotted in Excel (Figure 7).

**Table 9:** Primer sequences for qPCR analysis of Cse4, PolII and Ste12 ChIPs

**qPCR experiment**	**Pair name**	**Primer name**	**Sequence**
Cse4	Cse4P1	Cse4P1for	GATCAGCGCCAAACAATATGGAAAATCC
Cse4	Cse4P1	Cse4P1rev	AACTTCCACCAGTAAACGTTTCATATATCC
Cse4	Cse4P2	Cse4P2for	CGTATTACAATGGCCGAAGGC
Cse4	Cse4P2	Cse4P2rev	GCGACAACAAGAGGGAAATGA
Cse4	Cse4P3	Cse4P3for	CGTCCAAACATGAAAGTGCTCC
Cse4	Cse4P3	Cse4P3rev	CAGCGATTGACTTTCTCCCATT
Cse4	Cse4P4	Cse4P4for	GAAGCGTTATGGAACCTGTCGAA
Cse4	Cse4P4	Cse4P4rev	GTCGGTCGTCCAATATCATCGTAAA

PolII	Pol2P1	Pol2P1for	ACCGGTACAAGGACAAGACG
PolII	Pol2P1	Pol2P1rev	GTTCGTTCTCACGCACTTCA
PolII	Pol2P2	Pol2P2for	AAGACGCTCGAAACCAAGTG
PolII	Pol2P2	Pol2P2rev	GCTCACGTTTTGCAATGATG
PolII	Pol2P3	Pol2P3for	GACCGTTGCAAGGATTGATAA
PolII	Pol2P3	Pol2P3rev	TCAACCGAAGGAAGGAGAAA
PolII	Pol2P4	Pol2P4for	ATTGCCTGGTTCTTGTCCTG
PolII	Pol2P4	Pol2P4rev	CGTTGGCATATCACACCTTG

Ste12	Ste12P1	Pseudo35	CCCGTAGTCCGGTTTAATCA
Ste12	Ste12P1	Pseudo36	AACTGTGCATGAGCCAAGAG
Ste12	Ste12P2	Pseudo37	AAAAGGAGATAGGGCCCAGA
Ste12	Ste12P2	Pseudo38	CCAGAACAGCCAGCTAGACC
Ste12	Ste12P3	Pseudo39	TCGGGCTTCTAAGGCAAATA
Ste12	Ste12P3	Pseudo40	TCCTTTAAATGATGTTGCGATG
Ste12	Ste12P4	Pseudo41	TGTAGCCCAACGGATTCTTC
Ste12	Ste12P4	Pseudo42	AGAAGCTTTGCCAGGTGAAA

### Adapter design and annealing

Each barcode has a 3 base index followed by a 'T' (position 4) to promote pairing with the 'A'-overhang that is added to the samples as part of the Illumina genomic DNA library preparation. Barcodes were created in such a way that no barcode had the same base at positions 1, 2 and 3 for two reasons. First, we wanted to have a balanced base composition. Second, since ELAND alignment software allows 2 mismatches to map any read, we wanted to make sure that sequencing errors would not influence mapping. These four barcodes were used for this study: GTAT, CATT, ACGT and TGCT.

Oligos were synthesized by MWG at a 0.05 μmol scale with HPLC purification. Oligo sequences are given in Table 1. Each oligo was resuspended in annealing buffer (10 mM Tris [pH 7.5], 50 mM NaCl, 1 mM EDTA) to 200 μM. The forward and reverse oligos for each pair were mixed in equal volumes to 100 μM and denatured for 5 min at 95°C in a wet heat block. The heat block was then removed to room temperature and allowed to cool slowly over 45 min to promote annealing. For input DNA, Illumina genomic DNA adapters were diluted 1:20 using RNase-free DNase-free water (Gibco) and our adapters were diluted 1:30. For ChIP DNA libraries, the DNA concentrations of the barcoded-adapters were measured by a Nanodrop spectrophotometer and adjusted to the same working concentration as Illumina's adapters. The following dilutions were therefore determined: 1:500 for barcode GTAT, 1:450 for barcode CATT, 1:750 for barcode ACGT and 1:330 for barcode TGCT.

### Illumina DNA library generation

We followed the manufacturer's protocol for creating genomic DNA libraries with a few modifications based on experience gained from ChIP-Sequencing [6]. Here we present our modifications for generating barcoded libraries. ChIP DNA and input DNA were first band-isolated on a 2% agarose to obtain fragments between 150 and 350 base pairs and DNA was extracted using the QIAquick gel extraction kit (Qiagen) and eluted in 34 μL. For input DNA, because DNA amounts are higher than DNA recovered by ChIP, input DNA after gel extraction was diluted 1:5. After end-repair and addition of a single adenosine ("A") nucleotide, adapters were ligated to samples for 15 min at room temperature in the following fashion: the samples eluted from the MinElute column in 10 μL were ligated to 1 μL of adapters using 1.3 μL of LigaFast T4 DNA Ligase (3 Units/μL; Promega) and 12.3 μL Rapid Ligation Buffer (Promega). For input DNA libraries, the Illumina genomic DNA adapters were diluted 1:20 and all barcoded adapters were diluted 1:30. For ChIP DNA libraries, the Illumina genomic DNA adapters were diluted 1:40 and barcoded adapter dilutions are described in the previous section. After 15 min, samples were purified with the MinElute PCR purification kit (Qiagen).

We found that size-selection and purification of the ligation products by agarose gel before amplification of the library by PCR resulted in better libraries and decreased the incidence and intensity of an adapter-adapter band at ~120 bp. To eliminate those adapters that lack a fragment insert, samples were run on a 2% agarose E-Gel (Invitrogen) for 20 min, together with Track-It 50 bp DNA ladder (Invitrogen). DNA fragments ranging from 150 base pairs to 500 base pairs were extracted and recovered in 28 μL EB with a QIAquick gel extraction kit (Qiagen). To amplify the library, PCR was performed using Illumina genomic DNA primer "1.1" and Illumina genomic DNA primer "2.1" as described [6,62] with 15 cycles (Input DNA) or 17 cycles (ChIP DNA) of amplification. A final size selection was performed using a 2% agarose E-Gel to obtain a library with a median length of ~230 bp which is within the recommended size range for cluster generation on Illumina's flowcell. The library was recovered in 20 μL EB using MinElute Gel Extraction kit (Qiagen). Finally, DNA concentrations and purities (A_260/280 _nm ratios) were measured on a Nanodrop spectrophotometer and are given in Table 10.

**Table 10:** Experimental design and values for ChIP replicates and input samples

**Experiment #**	**Experiment ID**	**Barcode**	**Sample**	**DNA Concentration (ng/φL)**	**A_260/280_**	**Total reads**	**Mapped reads**	**% Mapping**	**Threshold**
**1**	PolII_Rep1	ACGT	PolII	16.6	1.83	1110177	623328	56.15	14
**2**	Input_CATT	CATT	Input	51.6	1.85	2526689	1719855	68.07	Not being scored
**3**	Cse4_Rep1	GTAT	Cse4	12.0	1.99	2606497	1080537	41.46	81
**4**	Ste12_Rep1	TGCT	Ste12	11.6	1.85	2020432	1072428	53.08	42
**5**	Input_ACGT	ACGT	Input	66.9	1.80	4678579	2857615	61.08	Not being scored
**6**	Cse4_Rep2	CATT	Cse4	8.6	1.85	679315	357384	52.61	25
**7**	Ste12_Rep2	GTAT	Ste12	9.4	1.86	2316023	1067739	46.10	44
**8**	PolII_Rep2	TGCT	PolII	15.5	2.00	3991833	2561147	64.16	12
**9**	PolII_Rep3	ACGT	PolII	9.7	1.97	2661304	1725793	64.85	42
**10**	Input_NB	None	Input	60.4	1.83	2432221	1582226	65.05	Not being scored
11A*	Cse4_Rep3	None	Cse4	10.7	1.69	1168583	239580	20.50	60
11B*	Cse4_Rep3	None	Cse4	10.7	1.69	3245287	583876	17.99	
12A*	Ste12_Rep3	None	Ste12	9.1	1.64	2106917	473965	22.50	39
12B*	Ste12_Rep3	None	Ste12	9.1	1.64	2668067	551040	20.65	
**13**	PolII_Rep4	None	PolII	18.8	1.94	4649077	3151068	67.78	40
**14**	Input_NB	None	Input	60.4	1.83	4373256	2455181	56.14	Not being scored
**15**	Input_ACGT	ACGT	Input	66.9	1.80	873037	529038	60.60	5
**16**	Input_CATT	CATT	Input	51.6	1.85	799430	479546	59.99	5
**17**	Input_GTAT	GTAT	Input	34.6	1.69	1380820	850262	61.58	6
**18**	Input_TGCT	TGCT	Input	68.1	1.78	611123	362458	59.31	4

NB (No Barcode) indicates that standard non-barcoded Illumina adapters were used. DNA concentrations of Illumina DNA libraries and their A_260/280 _ratios are given in columns 5 and 6.

Mapping values after sequencing (total sequencing reads, total mapped reads and percentage of mapped reads) are shown in columns 7, 8 and 9.

Threshold values for PeakSeq scoring algorithm are displayed in column 10.

* Sample ran twice on sequencer and reads pooled together from both lanes for scoring as a single experiment

### Mapping values and scoring

Raw data from the Illumina Genome Analyzer I and II were analyzed with Illumina's Firecrest, Bustard and GERALD modules for image analysis, basecalling and run metrics respectively, and a PhiX174 control lane was used for matrix and phasing estimations, as per the manufacturer's instructions. At this stage, a Perl script was used to partition the reads and remove the barcodes, i.e. the first four bases of each read. The next 26 bases of each read were aligned against the reference genome S288c *Saccharomyces cerevisiae*, using Illumina's ELAND program in standalone mode. For each barcode from each flowcell lane of barcoded libraries, the numbers of total and mapped reads were determined. Reads lacking a fully-intact barcode were discarded in a fifth bin called unclassified (not shown) and were not used in individual barcode mapping analysis, although they are calculated in the global lane mapping analysis. Mapping values and thresholds for scoring are given in Table 10. Uniquely-mapped reads with ChIP factors and barcoding schemes in common were pooled to produce 19 different data sets (Table 10). A full lane of non-barcoded input DNA comprising 2,455,181 mapped reads was used as a control when scoring barcoded input DNA. For scoring ChIP DNA relative to input DNA, a reference set consisting of two full lanes of input DNA and two barcoded input DNA data sets were combined, adding up to 13,198,172 total reads. Signal files were created using a 200 bp sliding window and scoring was performed with the PeakSeq program [63] using a mappability fraction of 1.0. Significant "ChIP hits" were determined relative to the corresponding input DNA by PeakSeq and further filtered by requiring a hit length of at least 100 bp, a p-value of < 0.05, a ratio of at least 2.0 between ChIP DNA and input DNA read counts and a difference of at least 10 between the ChIP DNA and input DNA read counts.

In the section "Similar barcode behavior with the same DNA sample", each barcoded input DNA (Table 10, experiments #15–18) was scored against the non-barcoded input DNA (Table 10, experiment #14) using PeakSeq. Similar criteria were applied to filter barcoded input DNA hits. In these significant "input hits" from all four barcoded input DNA samples, the number of overlapping nucleotides was determined. Finally, the total number of nucleotide positions from all significant "input hits" was calculated and compared to the *S. cerevisiae *genome size.

### Signal tracks and browser

Signal tracks were produced from uniquely-mapped reads and the *S. cerevisiae *genome using a 200 base pair sliding window. The Integrated Genome Browser (IGB, Affymetrix) was used to view images of signal tracks and to overlay them against the October 2003 version of the *Saccharomyces cerevisiae *genome. For ORFs and other annotations, the *Saccharomyces cerevisiae *genome was imported from SGD, the *Saccharomyces *Genome Database . After barcode parsing, each barcoded sample was compared over input DNA signal. To build signal tracks for comparing samples with different number of sequencing reads, the y-axis was normalized for each sample according to the total number of mapped reads.

### Comparisons of the average tag counts in 500 bp bins between two experiments

We divided the yeast genome into 500 bp bins. For each experiment (Table 10), we calculated the average tag count for each bin by summing the tag counts at each nucleotide position within a bin and dividing the total by 500. We then normalized each experiment by dividing the average tag count for each bin by the total tag count for the experiment. Normalized average tag count for each 500 bp bin were compared between two experiments among barcoded and non-barcoded samples for a particular factor. Linear regression was performed using R and the R^2 ^(R-squared) and p-values recorded.

### Target list annotation

For RNA PolII, target lists were overlapped with SGD ORFs and then annotated using SQL operation chains. Targets that did not overlap with any ORF were manually annotated to the closest gene by comparing PolII bed files in IGB. If we could not discriminate between two genes, then both were selected.

For Cse4, target lists were manually curated. CENs and ORFs were identified manually by looking at Cse4 signal tracks and Cse4 bed files in IGB and associating them to overlapping features. If one target overlapped two ORFs, both ORFs were included in subsequent analyses. If one target was located between two ORFs, the closest ORF was selected if the target's signal was noticeably higher towards this ORF and if the target was positioned closer to this ORF by at least 200 bp. Otherwise, both ORFs were considered.

For Ste12, target lists were manually curated. Targets located within 1.5 kb upstream of an ORF's transcription start site were assigned uniquely to that ORF. In the case of two ORFs with divergent promoters, both ORFs were considered if they were located less than 1 kb away from the target. For all other targets, the closest ORF was selected except when distances between a target and its two closest ORFs were less than 200 bp. In this case, both ORFs were included. Targets whose signals were marginally above background and that were distal to annotated regions of the genome were discarded.

### Comparisons between ChIP-Seq and published ChIP-chip data

Comparisons between PolII ChIP-Seq signal tracks and PolII ChIP-chip signal profiles around genes were made using data from Steinmetz et al. [54] and were performed visually in IGB. Comparisons between Ste12 ChIP-Seq targets and Ste12 ChIP-chip targets were done manually using IGB. If a ChIP-chip target was located less than 150 bp away from a ChIP-Seq target in at least one of the biological replicates, they were considered as overlapping for analysis purposes. ChIP-chip targets located more than 2 kb from a gene were excluded from comparisons. For Ste12, the Ste12 ChIP-chip target list from Borneman et al. [37] was used. For PolII ChIP-chip data from Steinmetz et al. [54], readily available on SGD and GEO (Series GSE6293, Sample GSM144667) [64], similar analyses and criteria as in Borneman et al. [37] were used to determine PolII ChIP-chip targets for consistency. 929 PolII ChIP-chip targets were visually inspected in IGB to ensure that they matched the PolII ChIP-chip signal profile [54]. Comparison with PolII ChIP-Seq data was done manually using IGB with similar criteria as those described for the Ste12 comparison between ChIP-Seq and ChIP-chip targets.

### Rank-rank correlations

In order to demonstrate that the rank-order lists of target binding sites for different samples are correlated, we performed the following comparisons. Lists of target binding sites are ranked first by p-value from most significant to least significant and in second by the difference between the ChIP DNA and input DNA read counts from the highest to the lowest. For a pairwise comparison of two target lists, we can vary the fraction of each list that is compared. The number of targets that agree (i.e. overlap by at least one bp) between the two lists can be plotted as a function of the fraction of the two lists compared. Ideally for targets that are identical the rank-rank plot will be linear having a value of 100% agreement when the entire rank lists are compared. Deviation for this linear correlation shows where the rank lists being compared begin to differ.

### Gene Ontology (GO) analysis

GO analyses were performed on SGD . We chose the GO Process Ontology with a p-value cutoff at p < 0.01. Other settings were set at default. For all three factors, ORFs were required to be present in at least one biological replicate. For Cse4 and PolII, all ORFs were used for the analyses. For Ste12, only those ORFs that were downstream of a target were analyzed.

### Simulation of depth of sequencing

To investigate the sequencing depth necessary to saturate the number of detectable binding sites, we performed the following computational simulation. We simulated a genome of size *S*_*G*_, with *N*_*sites *_binding sites of average fold enrichment *f*, using *N*_*reads *_mapped sequence reads. In order to perform the simulation efficiently, we scaled the genome size to 1 Mb, and correspondingly scaled the number of binding sites and mapped reads. We randomly generated sequence reads using a uniform distribution over the 1 Mb interval for both a simulated input DNA sample as well as for a ChIP DNA sample with regions with enriched numbers of sequence reads corresponding to binding sites. These simulated datasets were scored in the same manner as ChIP DNA data was scored against input using a false discovery rate of 5%. The sensitivity (i.e. the fraction of simulated binding sites identified) as well as the positive predictive value (i.e. the accuracy of the target sites identified) of the scored results was computed using the simulated data. The simulation was repeated 50 times in order to accurately estimate the sensitivity and positive predictive values. By varying the number of simulated mapped sequence-reads *N*_*reads*_, we can compute the minimum number of reads required in order to achieve a sensitivity of 95% (i.e. to be able to identify 95% of the simulated binding sites).

## Authors' contributions

The laboratories of MS and MG contributed to this publication. PL, GME, RKA, MS and CMY drafted the manuscript. GME, PL and MS designed the multiplexing scheme. PL performed benchwork experiments, target list analyses and comparisons. GME designed the barcoded adapters, performed Illumina sequencing and contributed to troubleshooting and analysis. RKA adapted PeakSeq for the barcoded yeast ChIP-Seq context, created a pipeline for barcoded ChIP-Seq, scored the data and contributed to troubleshooting. JR created the scoring algorithm (PeakSeq), the sequencing depth algorithm and performed the rank-rank correlations. TG performed sequencing depth simulations. CMY generated strains and contributed to data analysis. All authors read and approved the final manuscript.

## Supplementary Material

Additional file 1 **Cse4 target lists.** This data correspond to the annotated filtered target lists and the raw 'hits' returned from PeakSeq for three biological replicates of Cse4 ChIP DNA processed by ChIP-Seq. Click here for file

Additional file 2 **Ste12 target lists.** This data correspond to the annotated filtered target lists and the raw 'hits' returned from PeakSeq for three biological replicates of Ste12 ChIP DNA processed by ChIP-Seq. Click here for file

Additional file 3 **PolII target lists.** This data correspond to the annotated filtered target lists and the raw 'hits' returned from PeakSeq for four biological replicates of PolII ChIP DNA processed by ChIP-Seq. Click here for file

Additional file 5 **Complete GO analysis for Cse4, Ste12 and PolII.** This data represent the complete GO process analysis for Cse4 combined targets, Ste12 combined targets and PolII combined targets, with a p-value cutoff < 0.01. Click here for file

Additional file 4 **Sequencing artifacts present in non-barcoded samples and absent in barcoded samples.** These IGB signal tracks show abnormally-shaped peaks present in non-barcoded biological replicates at a particular genomic location that are absent in barcoded biological replicates. Click here for file
