# Supplementary material for: Efficient yeast ChIP-Seq using multiplex short-read DNA sequencing
Source: BMC Genomics. 2009 Jan 21;10:37. doi: 10.1186/1471-2164-10-37 (PMC2656530; doi:10.1186/1471-2164-10-37)
Supplement: Additional file 4 — Sequencing artifacts present in non-barcoded samples and absent in barcoded samples. These IGB signal tracks show abnormally-shaped peaks present in non-barcoded biological replicates at a particular genomic location that are absent in barcoded biological replicates. [file 1471-2164-10-37-S4.pdf]

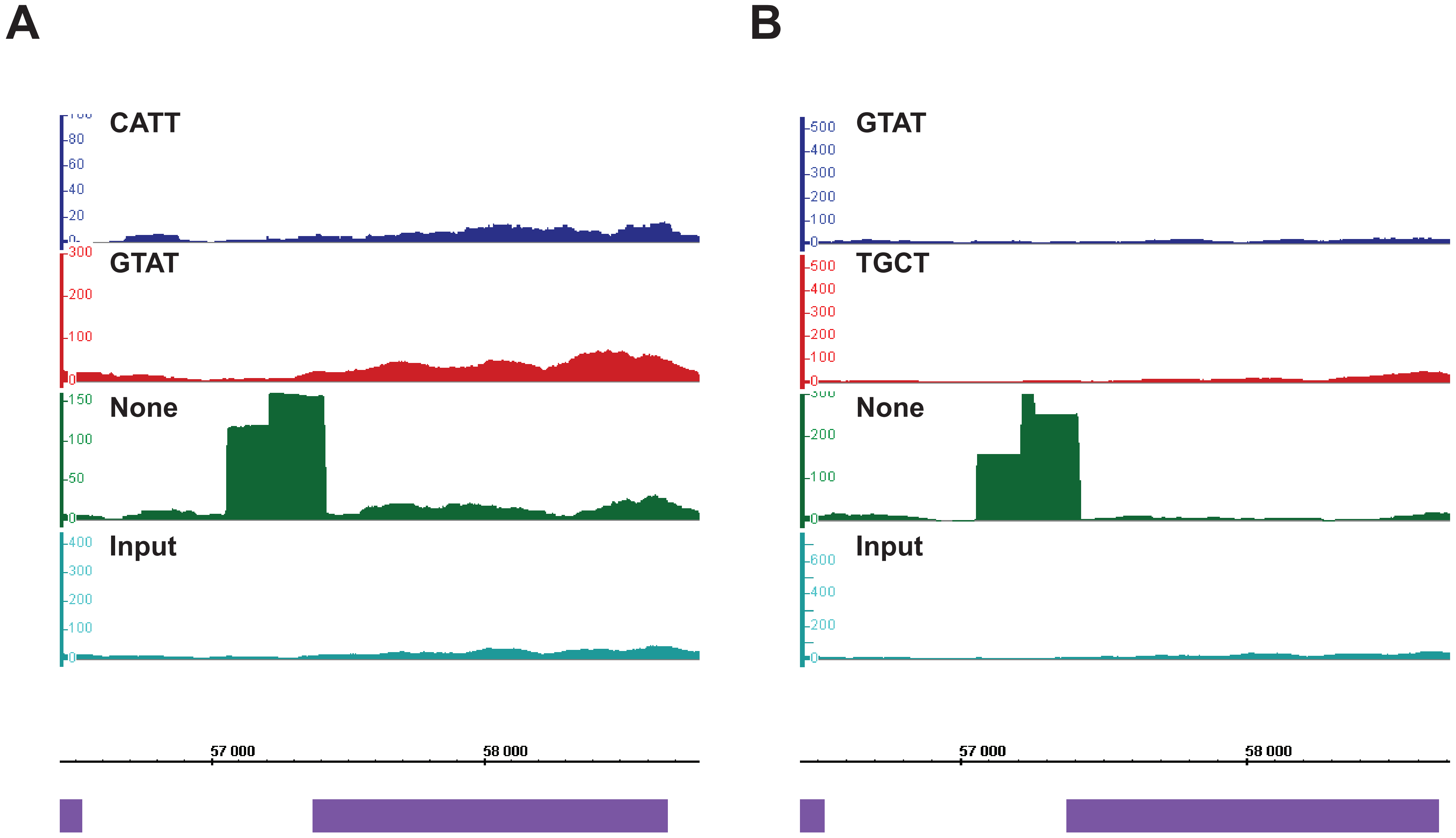

Additional File 4. Some sequencing artifacts found in non-barcoded samples disappear in barcoded samples. A series of few non-continuous reads highly represented gave this abnormal appearance peak in the non-barcoded signal profile (green), which indicates a false peak, similar to hybridization artifacts observed sometimes in ChIP-chip studies. (a) Cse4 displays an artifact at chromosome 3 between 57,000 and 58,000. Two barcoded replicates (Cse4_Rep2, dark blue; Cse4_Rep1, red) and one non-barcoded replicate (Cse4_Rep3, green) are compared to input DNA (light blue). The artifact is only present in the non-barcoded sample (green). (b) Ste12 displays an artifact at the same location as in (a). Two barcoded replicates (Ste12_Rep2, dark blue; Ste12_Rep1, red) and one non-barcoded replicate (Ste12_Rep3, green) are compared to input DNA (light blue). As in (a), the artifact is only present in the non-barcoded sample (green). For (a) and (b), IGB signal tracks of chromosome 3 between 56,000 and 59,000 are shown for each sample. Axis and scale normalizations are similar to Figure 2.
